# Genome Information of *Methylobacterium oryzae*, a Plant-Probiotic Methylotroph in the Phyllosphere

**DOI:** 10.1371/journal.pone.0106704

**Published:** 2014-09-11

**Authors:** Min-Jung Kwak, Haeyoung Jeong, Munusamy Madhaiyan, Yi Lee, Tong-Min Sa, Tae Kwang Oh, Jihyun F. Kim

**Affiliations:** 1 Department of Systems Biology, and Division of Life Sciences, Yonsei University, Seodaemun-gu, Seoul, Republic of Korea; 2 Korea Research Institute of Bioscience and Biotechnology, Yuseong-gu, Daejeon, Republic of Korea; 3 Biosystems and Bioengineering Program, University of Science and Technology, Yuseong-gu, Daejeon, Republic of Korea; 4 Department of Agricultural Chemistry, Chungbuk National University, Heungdeok-gu, Cheongju, Republic of Korea; 5 Biomaterials and Biocatalysts Group, Temasek Life Sciences Laboratory, National University of Singapore, Singapore, Singapore; 6 Department of Industrial Plant Science and Technology, Chungbuk National University, Heungdeok-gu, Cheongju, Republic of Korea; 7 21C Frontier Microbial Genomics and Applications Center, Yuseong-gu, Daejeon, Republic of Korea; Soonchunhyang University, Republic of Korea

## Abstract

Pink-pigmented facultative methylotrophs in the *Rhizobiales* are widespread in the environment, and many *Methylobacterium* species associated with plants produce plant growth-promoting substances. To gain insights into the life style at the phyllosphere and the genetic bases of plant growth promotion, we determined and analyzed the complete genome sequence of *Methylobacterium oryzae* CBMB20^T^, a strain isolated from rice stem. The genome consists of a 6.29-Mb chromosome and four plasmids, designated as pMOC1 to pMOC4. Among the 6,274 coding sequences in the chromosome, the bacterium has, besides most of the genes for the central metabolism, all of the essential genes for the assimilation and dissimilation of methanol that are either located in methylotrophy islands or dispersed. *M. oryzae* is equipped with several kinds of genes for adaptation to plant surfaces such as defense against UV radiation, oxidative stress, desiccation, or nutrient deficiency, as well as high proportion of genes related to motility and signaling. Moreover, it has an array of genes involved in metabolic pathways that may contribute to promotion of plant growth; they include auxin biosynthesis, cytokine biosynthesis, vitamin B_12_ biosynthesis, urea metabolism, biosorption of heavy metals or decrease of metal toxicity, pyrroloquinoline quinone biosynthesis, 1-aminocyclopropane-1-carboxylate deamination, phosphate solubilization, and thiosulfate oxidation. Through the genome analysis of *M. oryzae*, we provide information on the full gene complement of *M. oryzae* that resides in the aerial parts of plants and enhances plant growth. The plant-associated lifestyle of *M. oryzae* pertaining to methylotrophy and plant growth promotion, and its potential as a candidate for a bioinoculant targeted to the phyllosphere and focused on phytostimulation are illuminated.

## Introduction

Alphaproteobacteria of the genus *Methylobacterium* are pink-pigmented facultative methylotrophs capable of growth on one-carbon (C_1_) compounds, such as methanol and methylamine, as well as on a variety of C_2_, C_3_, and C_4_ compounds [1], [2]. Members of this genus in the family *Methylobacteriaceae* that belongs to the order *Rhizobiales* are ubiquitous in nature. They have been detected in soil, dust, freshwater, lake sediments, the air, and especially plants [3], [4]. In association with plants, *Methylobacterium* species may colonize the phyllosphere, the rhizosphere, or the intercellular spaces (also called the endosphere) [5]–[7]. The importance of *Methylobacterium* in the plant phyllosphere has been well documented, and *Methylobacterium* can benefit from methanol that is released by the plant [8], [9].

Methanol, which is emitted by the plant as a result of cell-wall degradation, is one of the main C_1_ carbon sources to *Methylobacterium* [10], [11]. It was estimated that *Methylobacterium* living in plants can reduce methanol emission significantly [12]. The terrestrial leaf surface area that microorganisms can colonize is about 6.4×10^8 ^km^2^ [13], and methanol with its concentration of 100 Tg y^−1^ emitted by the plants is the third most abundant organic compound in the atmosphere [11]. Therefore, the genus *Methylobacterium* is expected to serve important roles in circulation of carbon and energy in the ecosystem. The C_1_ metabolism of *Methylobacterium* appears to enable them to out-compete against other phyllosphere microorganisms [8]. Furthermore, recent studies reveal that *Methylobacterium* was detected in extreme environments such as Antarctic soil [14] and biological soil crusts [15] at a high proportion.

A number of *Methylobacterium* species are well-known to produce plant growth hormones, and can be classified as plant growth-promoting bacteria [1], [16], [17]. They can be categorized into three types based on their underlying mechanisms. The first is as a bio-stimulator that directly influences plant growth by producing plant growth hormones. The second is as a bio-fertilizer that may provide micronutrients such as nitrogen and phosphate to the plants. The third is as a bio-controller that can suppress the growth of pathogens by producing anti-fungal metabolites and can induce induced systemic resistance in plants [18]. Among the fifty-some *Methylobacterium* type species that have been isolated from diverse environments, complete genome sequences of eight strains out of six species are available [10], [19].

*Methylobacterium oryzae* CBMB20^T^, isolated from the surface of the stem of rice (*Oryza sativa* L. cv. Nam-Pyeoung) [20], is a plant growth-promoting methylotroph that has been reported to produce auxin, cytokinin, and 1-aminocyclopropane-1-carboxylate (ACC) deaminase [21]–[25]. Phylogenetic analysis based on the 16S ribosomal RNA sequence revealed that it is highly similar to *Methylobacterium radiotolerans*, a species isolated from rice, too [20]. Despite the multifaceted potential of *M. oryzae* in agriculture and bioremediation, its molecular basis in light of genome information has not been seriously explored. Specifically, there are a multitude of experimental evidence for various factors involved in the symbiotic relationship between *Methylobacterium* species and the plants [22], [26]–[28], but their genetic foundation has not been clearly revealed.

In this study, we determined the complete genome sequence of *M. oryzae* CBMB20 that had been isolated from the phyllospheric environment. Detailed genome analysis was entailed to disclose the generic and specific features of the genetic information. From the genome, we detected several kinds of genes for adaptation to the aerial parts of plants or plant growth promotion.

## Materials and Methods

### Genome Sequencing and Assembly

Genomic DNA was isolated from a 48-hour culture of *M. oryzae* strain CBMB20 (DSM 18207 = LMG 23582 = KACC 11585) by the use of DNeasy blood and tissue kit (Qiagen). Whole-genome sequencing of the bacterium was carried out using Roche’s 454 pyrosequencing technology. 1,861,429 shotgun reads (781.6 Mb, ca. 120×genome coverage) produced from one and a half of a full plate run using GS FLX Titanium and 3,510 fosmid end reads (average 576.0 bp/read) were assembled into 82 large contigs (>500 bp, totaling 6.39 Mb) in 30 scaffolds by gsAssembler (National Instrumentation Center for Environmental Management [NICEM], Republic of Korea). Gaps were closed by either primer walking with gap-spanning PCR products or manual joining of overlapping reads protruding from the ends of adjacent contigs. After the gap closure, accuracy of the overall assembly was validated by checking the consistency of fosmid paired-end reads. An over-collapsed region with an extraordinarily high read depth that covered fosmid clones whose apparent clone sizes are shorter that the average value was re-sequenced as follows: plasmid shotgun library was constructed from the three fosmid clones covering the repeat-induced over-collapsed region, and the resulting Sanger pair-ended reads were assembled by using Arachne [29]. Remaining gaps were joined using 454 reads extracted from the relevant region.

### Genome Annotation and Computational Analysis

Protein-coding genes and tRNA genes were predicted by Glimmer [30] and tRNAscan-SE [31], respectively. rRNA genes were identified by searching genes from other sequenced *Methylobacterium* species by BLASTN. Functional annotation was performed by AutoFACT [32] that assigns informative hit description hierarchically from the results obtained by BLASTP search against UniRef90, KEGG genes, COG, and NCBI NR databases. Additionally, TIGRFAMs hits over trusted cutoff value were given the highest priority. Chromosomal sequence was also subject to the RAST server [33] for the automatic gene prediction and functional annotation. Translational start sites, the authenticity of the overlapping genes, and product names were manually validated by comparing these two annotation result in Artemis [34] and comparing with blast results against other *Methylobacterium* genome.

### Identification of Genomic Islands and CRISPRs

Genomic islands―regions potentially acquired through horizontal gene transfer―were identified in IslandViewer [35] which is a web-based island prediction tool and provides the integrated result of genomic islands from three different prediction tools: IslandPath-BIMOB [36], SIGI-HMM [37] and IslandPick [38]. For the genomic island prediction, IslandPath-BIMOB uses GC content, dinucleotide bias and location of mobile elements and SIGI-HMM uses codon usage of each genes and IslandPick uses comparative genomics approach. CRISPR candidates were identified with the CRISPRfinder tool (http://crispr.u-psud.fr/Server/) [39], and by structural analysis and comparison with those in the database.

### Genomic Comparison

The genome sequences of Methylobacterium extorquens AM1 (CP001510), M. extorquens CM4 (CP001298), M. extorquens DM4 (FP103042), M. extorquens PA1 (CP000908), M. extorquens BJ001 (CP001029), Methylobacterium nodulans ORS 2060 (CP001349), M. radiotolerans JCM 2831 (CP001001), and Methylobacterium sp. 4–46 (CP000943) were downloaded from GenBank. Homology searches were conducted against the genomes and plasmid sequences of close relatives at the nucleotide and amino acid level by using the BLAST software package. Core genome and pan-genome of Methylobacterium were defined using OrthoMCL after all-vs-all blast with the amino-acid sequences of each gene in the genomes of completely sequenced species. The calculation of ortholog groups was conducted using blast results with the cutoff value of 70% identity and 70% coverage.

### Phylogenetic Analysis

A phylogenetic tree was generated by molecular evolutionary genetic analysis (MEGA 5) [40] using the neighbor-joining method and the evolutionary distances were calculated using Jukes-Cantor method. Thirty six genes that are broadly conserved in the three domains of life [41] were used for the tree construction of the completely sequenced *Methylobacterium* species.

## Results and Discussion

### General Features

**Figure 1:**
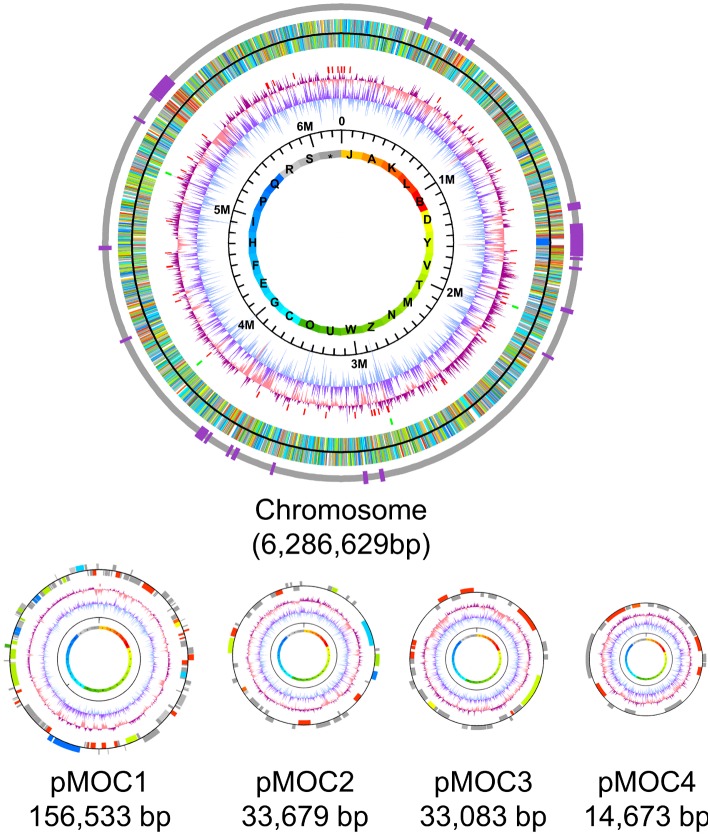
Circular representation of the five replicons of the *Methylobacterium oryzae* CBMB20^T^ genome. The first circle from inside is the color-coded COG functional category and next black circle indicates the scale bar. The third and fourth circles indicate the GC skew and the GC content. Next vertical red bars denote tRNAs and the green bars the rRNA operons. In the next circle are predicted CDSs represented by 25 colors according to COG functional classification (Table S4), and outermost bars indicate genomic islands (Table S1).

The genome of CBMB20, the type strain of the alphaproteobacterium *M. oryzae*, is comprised of a single circular chromosome of 6,286,629 bp with an overall G+C content of 69.8 percent and four plasmids, designated as pMOC1 (156,533 bp), pMOC2 (33,679 bp), pMOC3 (33,083 bp) and pMOC4 (14,673 bp), having an overall G+C contents of 63.6%, 60.6%, 64.0%, and 62.3%, respectively (Figure 1). The putative origin of replication was located at the upstream of the *dnaA* gene (MOC_0001) encoding a chromosomal replication initiation protein. *M. oryzae* chromosome encodes 6,274 coding sequences (CDSs); 4,152 (66.18%) CDSs are assigned putative biological functions (Table 1). There are a total of 58 tRNAs (one in pMOC1) and four ribosomal RNA operons. Chromosomally encoded genes for RNA polymerase and DNA polymerase I, III, IV were identified, whereas genes encoding DNA polymerase V subunits C and D were detected in pMOC1 (pMOC1_0006 and pMOC1_0007). There are 12 genes encoding resolvase domain-containing proteins (6 on chromosome, 6 on plasmids). *M. oryzae* has 20 genes encoding sigma factors: 15 are the *rpoE* family members, two are *rpoH* (MOC_4234 and MOC_6224), two are *rpoN* (MOC_2469 and MOC_2862), and one is *rpoD* (MOC_0735). 258 genes encode the transcriptional regulators that include 45 of the LysR family, 27 of the AraC family, 25 of the TetR family, 22 of the GntR family, and 17 of the LuxR family. There are 32 genes encoding aminoacyl-tRNA synthetases.

**Table 1:** General features of the *Methylobacterium oryzae* CBMB20^T^ genome.

	Chromosome	pMOC1	pMOC2	pMOC3	pMOC4
**Size (bp)**	6,286,629	156,533	33,679	33,083	14,673
**G+C content (%)**	69.77	63.63	60.57	64.04	62.3
**Number of CDSs**	6,274	141	32	36	18
**CDS summary**					
** Protein of assigned function**	4,152	87	18	16	7
** Protein of unassigned function**	2,122	55	14	20	11
** Not in COG**	2,375	83	20	24	13
**Percentage coding**	85.61	72.84	59.47	50.51	61.36
**Average gene length (bp)**	856	802	626	464	500
**tRNA**	57	1	-	-	-
**rRNA operon**	4	-	-	-	-
**GenBank accession number** *	CP003811	JX627580	JX627581	JX627582	JX627584

-, None identified.

*, Sequence information is also available at the Genome Encyclopedia of Microbes (GEM) homepage (http://www.gem.re.kr/).

In the *M. oryzae* CBMB20 chromosome, 25 genomic islands (522,562 bp, 8.31% of the entire chromosome) were detected (Table S1). Among the 510 protein-coding genes located in the genomic islands, 220 genes encode proteins of unassigned function and 88 genes encode phage and transposase related proteins. 111 genes encoding transposon-related proteins were identified in the chromosome; numbers of the frequently found ones include 23 of the IS*3* family, 14 of the IS*3*/IS*911* family, 11 of the IS*3*/IS*911* family, 10 of the IS*66* family, and 9 of the IS*116*/IS*110*/IS*902* family. Clustered regularly interspaced short palindromic repeat (CRISPR) element is a widely found defense mechanism of prokaryotes against foreign plasmids and phages [42]. In the CBMB20 chromosome, two CRISPRs were detected. The sizes of crispr1 and crispr2 are 305 bp and 248 bp, respectively, and each has three spacers. Four spacer sequences have homology with those of *M. radiotolerans* but the e-value is high; two in crispr1 have no homology with any sequences in the NCBI database.

### Energy Metabolism and Methylotrophy

Species of *Methylobaterium* are facultative methylotrophs that can use reduced C_1_ compounds such as methanol and methylamine, besides multi-carbon compounds, as a sole carbon source. *M. oryzae* may produce pyruvate from Fructose-6-phosphate through the pentose phosphate pathway or the Entner-Doudoroff pathway; the *pfk* gene encoding phosphofructokinase, one of the key enzymes in the glycolytic pathway, was not detected in the *Methylobacterium* genome. The C_1_ assimilation pathway can be divided into the serine pathway and the ribulose monophosphate pathway [43]. *Methylobacterium* assimilates single-carbon compounds through the serine pathway. Most of the genes related to the C_1_ assimilation of *Methylobacterium* were identified through the genetic studies of *M. extorquens* AM1, whose genome has been completely determined. Most of the genes related to methylotrophy are located in several large clusters [44]–[47].

**Figure 2:**
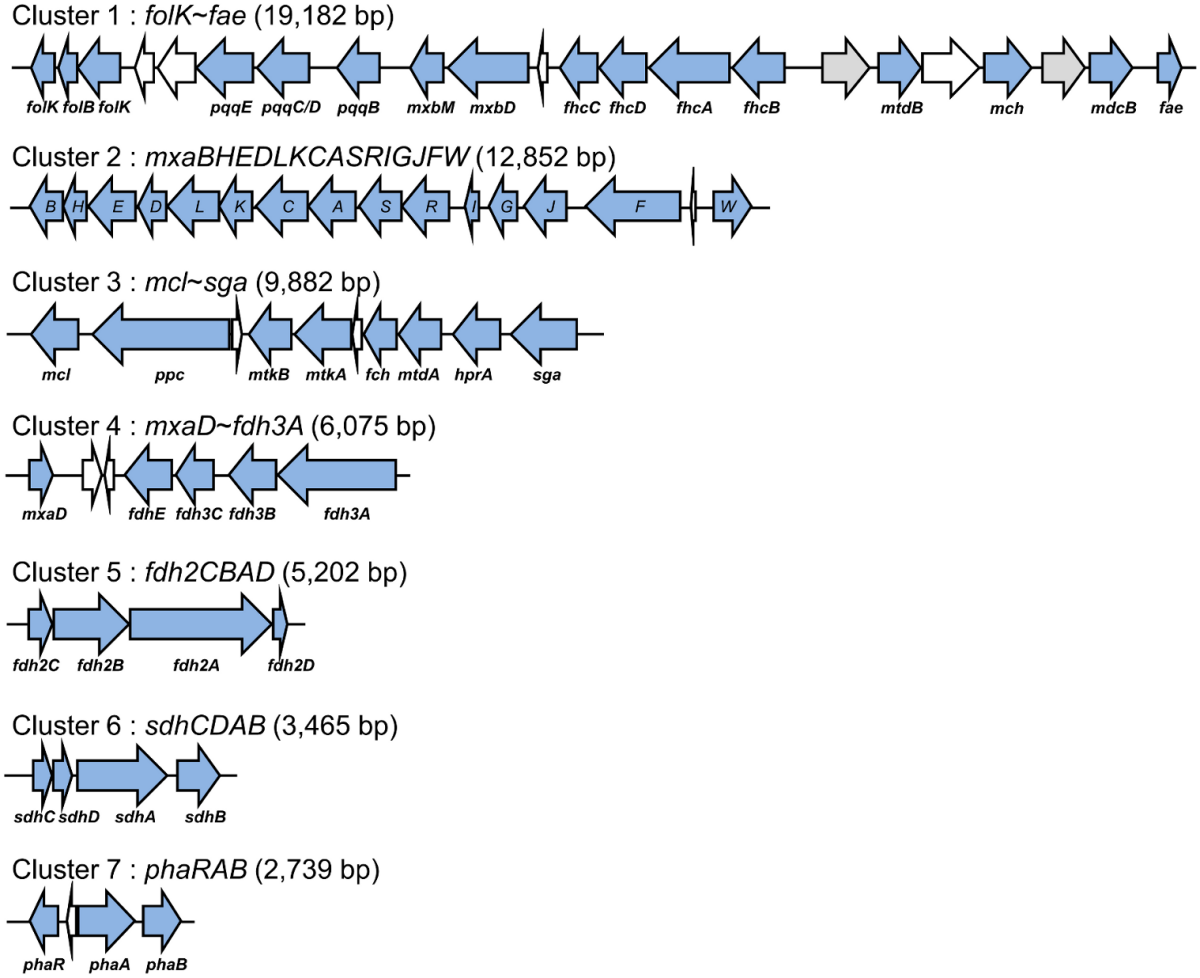
Methylotrophy gene clusters of *M. oryzae* CBMB20. The genetic loci for methylotrophy, surmounting to ca. 53 kb, are dispersed in the chromosome into seven clusters ranging from 2.74 to 19.18 kb in size and dozens of isolated genes. Gray arrows indicate the genes unrelated to methylotrophy. White arrows indicate the genes with unassigned function.

*M. oryzae* has more than 87 genes encoding methylotophy-related proteins, which cover primary oxidation of methanol, tetrahydromethanopterin-dependent formaldehyde dissimilation pathway, tetrahydrofolate-dependent C_1_ transfer pathway and serine cycle and form seven interspersed-methylotrophy clusters ranging in size from 2,739 bp to 19,182 bp (ca. 53 kb in total). They accommodate 55 methylotrophy genes, and the rest are distributed in the genomic regions between these clusters (Figure 2 and Table S2). One characteristic of the microorganisms that use the serine pathway to assimilate a single carbon is that the *aceA* gene encoding isocitrate lyase is absent. Isocitrate lyase is involved in the production of glyoxylate, an intermediate of the serine pathway, so *Methylobacterium* should make glyoxylate through the ethylmalonyl-CoA pathway [45]. Genes related to the ethylmalonyl-CoA pathway were also detected in methylotrophy islands.

Many *Methylobacterium* species have a number of genes related to photosynthesis. In CBMB20, genes encoding the light harvesting protein, the photosynthetic reaction center protein, and the bacteriochlorophyll biosynthesis protein are clustered in two loci. Moreover, these photosynthetic gene clusters are conserved in other *Methylobacterium* spp. (Figure S1). Indeed, these photosynthesis-related genes are up-regulated in specific conditions such as a methanol-containing medium in *M. extorquens* AM1 [48], and the bacteriochlorophyll of *Methylobacterium* was detected in in the phyllosphere [49] and biological soil crusts [15]. In addition, *M. oryzae* has 5 genes encoding the blue light receptor proteins (MOC_0547, MOC_2982, MOC_4577, MOC_5080 and MOC_6124).

### Adaptation to Plant-Associated Environments

The aerial part of plants such as the phyllosphere can be a challenging environment to microorganisms, because of UV irradiation, rapid change of temperature, water stress, and deficiency of available nutrients or limited access to nutrients (i.e. oligotrophic) [50]–[52]. *Methylobacterium* is known to colonize mainly around stomata or trichomes, or in intercellular spaces, which implies that these are better habitats for nutrient acquisition from plant tissues and avoidance of the environmental stresses [5],[6],[8],[53]. Genome analysis of CBMB20, an inhabitant of the rice stem, reveals genetic repertoire for adaptation to these environments.

#### UV irradiation and oxidative stress

UV irradiation is one of the severe stresses to phyllosphere microorganisms. The pink color of *Methylobacterium* results from the production of carotenoids [54], which may protect the microorganisms against the photo-oxidative stress and they conduct light harvest in photosynthetic bacteria. Many radiotolerant microorganisms isolated from the phyllosphere form pink to red colonies [13]. Like *M. extorquens* AM1 [55], *M. oryzae* has eight genes encoding carotenoid biosynthetic proteins―*crtB* (MOC_2698 and MOC_3207), *crtF* (MOC_3185), *crtE/I* (MOC_3187, MOC_3208, MOC_5734 and MOC_5741), and *crtC* (MOC_3188)―and the gene encoding a UV damage repair endonuclease (MOC_2537).

Plants use a variety of non-specific tactics to defend themselves against bacterial, viral and fungal threats, which include the production of reactive oxygen species (ROSs; superoxide, hydroperoxyl radical, hydrogen peroxide, and hydroxyl radical species), nitric oxide, and phytoalexins [56], [57]. Aerobic organisms should cope with the oxidative stress resulting from ROSs, harmful byproducts of the aerobic respiration, by means of several kinds of antioxidants and enzymes [58], [59]. In addition, the CBMB20 genome encodes genes to protect itself from these plant defense mechanisms. The genome has two genes encoding glutathione peroxidases (MOC_1064 and MOC_4484), one encoding a glutathione-disulfide reductase (Gor; MOC_3063), and two encoding superoxide dismutases (SOD; MOC_4362 and MOC_5157). Moreover, the bacterium has 10 genes encoding several kinds of peroxidases (9 on chromosome and 1 on pMOC4) and 11 genes encoding catalases. In addition, genes encoding four glutaredoxins (GrxC; MOC_0043, MOC_1346, MOC_1829 and MOC_2948), one peroxiredoxin (MOC_3773), three thioredoxins (MOC_1047, MOC_3885 and MOC_4677), and three redoxin domain-containing proteins (MOC_2186, MOC_2972 and MOC_3340) were identified.

#### Water stress and nutrient deficiency

The surface of the plants is a dry environment for microorganisms because it is directly exposed to sunlight and air, and available nutrients exist at very low concentrations and tend to localize in specific regions [60]. Microorganisms in the phyllosphere make extracellular polysaccharides to protect themselves from such stresses [5]. CBMB20 has 33 genes encoding polysaccharide-biosynthetic proteins, including nine lipopolysaccharide biosynthetic genes and five genes encoding the O-antigen polymerase. It was reported that an O-antigen deficient mutant of lipopolysaccharides diminishes the colonization ability in the rhizosphere [61]. The bacterium has three genes encoding trehalose biosynthesis proteins (MOC_1982, MOC_1984 and MOC_1985). The disaccharide trehalose is one of the most common compounds in nature for tolerance to desiccation. The trehalose prevents water loss and desiccation, and protects the cell from extracellular environments by making a gel form on the outside of cell [62]. It also has the *hpn* genes (*hpnHNKJI,* MOC_1185, MOC_1187 and MOC_1189 to MOC_1191) encoding hopanoid biosynthesis proteins. Hopanoids are pentacyclic compounds that are similar to eukaryotic cholesterol in terms of their structure and function. Indeed, there is a report on the production of hopanoids by *Methylobacterium* [63]. They are known to regulate the membrane fluidity and to adjust the membrane permeability, so it helps the adaptation to extreme extracellular environment [64].

#### Cold stress and cold shock proteins

The cold shock protein (Csp) family consists of small, highly conserved, and structurally related nucleic acid-binding proteins that presumably have important roles in regulation of various microbial physiological processes [65]. These proteins are widely distributed among prokaryotes, and are often encoded through differentially regulated multiple gene families [66]–[68]. Csps are thought to serve as nucleic acid chaperones that bind to RNA or DNA, and thus may facilitate the control of processes such as replication, transcription, and translation within bacterial cells [65]. Five *csp* genes (*cspA*, MOC_0360 and MOC_5949; *cspB*, MOC_3103; *cspC*, MOC_0148; and *cspG*, MOC_5724) are found in CBMB20 and have been linked to modulation of cold adaptation functions.

#### Motility and adhesion

It has been demonstrated that motility is a crucial factor for bacterial colonization in the phyllosphere with an experiment using a nonmotile *Pseudomonas* strain [61], [69], [70]. CBMB20 appears to have two sets of flagella-related genes, one in a single gene cluster and the other in at least four clusters, and 67 CDSs have been annotated as such. Most of the genes are in duplicate and some have three or four copies. In addition, there are 64 genes encoding chemotaxis-related proteins including 51 of methyl-accepting chemotaxis sensory transducers in the chromosome.

Type IV pili, displayed by a wide variety of Gram-negative bacteria, have a distinctive method of assembly involving proteolytic processing and N-methylation of the pilin precursor. Although components of type IV pili can be involved in adhesion to surfaces and are among the few factors known to affect endophytic colonization. The establishment of microcolonies on roots and fungal mycelium, and the systemic spreading in rice are also mediated by type VI pili [71]. In the CBMB20 genome, ten genes involved in type-IV pilus biosynthesis were identified. Two genes related to hemolysin-type adhesins are also present in the genome. A large number of genes coding for such proteins have been found in plant-associated bacteria [72]. The products of these genes may be required for surface attachment and biofilm formation during plant tissue colonization [73].

#### Signaling and gene regulation

Quorum sensing is a cell density-dependent response mechanism and many *Methylobacterium* spp. have this system [74], [75]. CBMB20 has one gene encoding autoinducer synthetase (MOC_6267), which shows 57% amino acid sequence identity (95% coverage) with the *mlaI* gene product of *M. extorquens* AM1 [76], three genes encoding luciferase-like monooxygenases (MOC_0369, MOC_2190 and MOC_4883), and 17 genes encoding the LuxR family of transcriptional regulators (17 in chromosome, 1 in pMOC1). In addition, a number of genes encoding homoserine modification enzymes that modify O-acetylhomoserine (MOC_0869) and O-succinylhomoserine (MetB, MOC_5078) were detected in the chromosome.

Two-component systems are the signaling pathways that respond to cues of extracellular environments. The two-component systems consist of a sensor protein that is a membrane-bound histidine kinase and phosphorylated by extracellular stimulation, and a receiver protein that induces intracellular responses after receiving the phosphoryl group from the sensor protein [77], [78]. CBMB20 has 86 genes encoding histidine kinases and 40 genes encoding receiver proteins and at least 35 genes encoding two component transcriptional regulators in the chromosome.

PhyR has a central role in the adaptation of *Methylobacterium* to the plant environment [9]. The PhyR regulon suggests a role in dealing with the various stresses that the bacteria are likely to encounter in the phyllosphere and this regulator is also importance in other alphaproteobacteria. PhyR contains both a receiver domain, which is normally found in two-component regulatory systems and a domain that resembles an extracytoplasmatic function (ECF) sigma factor [79]. In addition, the genome has RNA polymerase genes (*rpoD*, MOC_0735; *rpoH*, MOC_4234 and MOC_6224) to transcribe stress genes and 15 *rpoE* genes encoding the ECF subfamily of sigma factors.

#### Transport and secretion systems

The genome has 536 genes encoding transporter-related proteins. They include more than 30 genes encoding amino-acid transporters and a number of genes encoding several kinds of saccharide transporters. In addition, 28 genes encoding porins (25 on chromosome, two on pMOC1, and one on pMOC3) including six for carbohydrate selective porins were detected. It has 59 genes encoding the major facilitator superfamily of permeases and 24 genes encoding the RND family of efflux transporter MFP subunit proteins, and 21 genes encoding urea ABC transporters. It also has genes encoding the TolQRA-pal transporter system and the TonB-Exb transporter system. The TonB-dependent transport system has high affinity with specific substrates which exist in low concentrations or have low membrane permeability. Therefore, TonB-dependent transporters are important for uptake of specific substrates [80]. CBMB20 has more than 33 genes encoding tripartite ATP-independent periplasmic transporter proteins. This transport system is important for phytosymbionts, because C_4_-dicarboxylate such as malate is one of the major nutrients that can be acquired from the phyllosphere [81]. Also, three genes encoding a bacteriocin/lantibiotic ABC transporter and three genes (MOC_4802, MOC_4924 and MOC_5803) encoding auxin efflux carriers were identified. In the genome, 32 genes encoding secretion system proteins were identified, and they are involved in type I secretion, Sec-dependent transport, and twin-arginine translocation.

#### Pectin acetylesterase

*M. oryzae* uses methanol as well as sugars or amino acids emitted by the plants as a carbon source. Methanol, a by-product of the plant cell-wall metabolism [11], is emitted by the action of pectin methylesterase. CBMB20 has a gene encoding a putative pectin acetylesterase that hydrolyzes acetyl ester of pectin. As a result of deacetylation of pectin, the accessibility of methyl esterase and other enzymes such as rhamnoglacturonase and polygalacturonase to the pectin can be improved [82]. This suggests that CBMB20 can actively acquire the nutrients from the plants, not just passively receives them. Indeed, Jourand and colleagues observed that the pectin is highly disorganized in the *Methylobacterium*-existing regions [83].

#### Antimicrobial compounds

The CBMB20 genome has genes (MOC_0382, MOC_1520, MOC_1521 and MOC_1522) encoding a bacteriocin and an accompanying transport system that may inhibit the growth of the some pathogenic microbes [84]. It also has a gene (*ubiA*, MOC_5953) for 4-hydroxybenzoate polyprenyl transferase. A precursor of the important electron carrier ubiquinone, 4-hydroxybenzoate, is also known to have the antimicrobial activity. It has been reported that 4-hydroxybenzoate also stimulates the production of pathogen-related proteins in *Nicotiana tabacum*, although to a considerably lower extent than salicylic acid [85]. Concerning antibiotic resistance, the CBMB20 genome encodes a chloramphenicol acetyltransferase (MOC_0656) and the CBMB20 strain is resistant to chloramphenicol according to the original description of the strain [20]. CBMB20 has two genes encoding the syringopeptin synthetase C-related non-ribosomal peptide synthetase (MOC_1555 and MOC_1556) in the chromosome and one in pMOC1 (pMOC1_0073). Syringopeptin is another class of lipopeptide toxins [86].

#### Urea metabolism

The genus *Methylobacterium* was mainly isolated from phyllosphere, but they were also isolated from diverse environment including rhizosphere [53]. Although CBMB20 was isolated from phyllosphere, this bacterium is capable of synthesizing and degrading urea [20]. A complete set of genes were found that code for enzymes in the complete urea cycle. In this bacterium, arginine is probably synthesized using proline and carbamoyl phosphate as precursors. Urea is degraded by urease. The urease operon contains the structural genes *ureAB* (MOC_3228) and the accessory genes *ureD, ureE, ureF*, and *ureG* (MOC_2901, MOC_3230, MOC_3231 and MOC_3232). This operon is very similar to that of *Herbaspirillum seropedicae* and *Corynebacterium glutamicum* [87]. In addition, the genome of *M. oryzae* has 21 genes encoding components of the urea ABC transporters.

#### Heavy metal resistance

Some *Methylobacterium* strains have been described to metabolize a range of the toxic organic chemicals and toxic explosives [88], [89]. Moreover, it was found to be among the dominant genera in the rhizosphere and plant tissues of *Thlaspi geosingense*, a Ni hyperaccumulating plant [90], and have shown resistance to extremely high concentrations of heavy metals like cadmium, chromium, mercury and lead [91]. Heavy metals are also important cofactors, and the CBMB20 genome encodes several genes involved in heavy metal uptake and efflux. Genes were found for ABC transporters involved in the uptake of zinc (MOC_1734 and MOC_1736) or nickel (MOC_1821, MOC_2663 and MOC_2939). Nickel is an essential cofactor for urease [92]. In addition, the bacterium has 17 genes encoding TonB-dependent siderophore receptor proteins.

The CBMB20 genome carries genes putatively involved in copper resistance, including a copper-translocating P-type ATPase (MOC_4108) whose expression is regulated by CueR (MOC_4107) and the multiple copper oxidase CueO (MOC_5843). Indeed, CBMB20 exhibited the normal growth in a medium containg up to 4 mM copper (Figure S2). The genome also encodes an arsenic/arsenate resistance cluster in the chromosome (ArsCRH, MOC_4758 to MOC_4761). Other heavy metal resistance genes located in the chromosome include a putative chromate transport protein (ChrA, MOC_1822) and a cation efflux system protein CzcA (MOC_1814) involved in cobalt-zinc-cadmium resistance. CBMB20 is able to tolerate up to 3 mM NiCl_2_/CdCl_2_ [91].

### Promotion of Plant Growth

Plant growth-promoting bacteria can benefit plants in a number of ways [18]. Many of them produce phytohormones or several kinds of enzymes which can influence to plant growth. They may provide the plants with a nitrogen source by fixation of atmospheric nitrogen to ammonia, or other nutrients such as phosphate, sulfur or iron. Further, they may even protect the plants from plant pathogens by suppressing the growth of the pathogens. Many of the *Methylobacterium* species are known to influence plant growth through the production of plant hormones or enzymes [93]. *M. oryzae* is known to produce auxin, cytokinin and ACC deaminase [22]–[24].

#### Phytostimulation

Some strains of *Methylobacterium* including CBMB20 are known to produce auxins [25]. Currently, there are three major auxin-biosynthetic pathways [94]. The KEGG pathway database presents one complete auxin biosynthesis pathway from *M. nodulans*. In this pathway, indole-3-acetic acid is produced from tryptophan through tryptamine and indole-3-acetaldehyde. This pathway is composed of tryptamine decarboxylase, amine oxidase, and aldehyde dehydrogenase. CBMB20 has genes encoding amine oxidase (MOC_0996, MOC_3058 and MOC_3474) and aldehyde dehydrogenase (MOC_0153, MOC_0181, MOC_0592, MOC_2514, MOC_5534 and MOC_6112). However, the gene encoding tryptamine decarboxylase was not detected. This enzyme is aspartate aminotransferase superfamily protein that contains pyridoxal phosphate-binding site and also, called aromatic-L-amino acid decarboxylase. As CBMB20 has genes encoding aspartate aminotransferase (MOC_2191) and aromatic amino acid *β*-eliminating lyase/threonine aldolase (MOC_0009), we assume that one of them may serve a function similar to that of tryptamine decarboxylase.

Zeatin is a major cytokinin and is produced by two pathways [95]. One is *de novo* synthesis that the zeatin is produced from adenosine monophosphate by the action of adenylate isopentenyltransferase, cytokinin trans-hydroxylase, and several hydrolase. The other is that the zeatin is produced through isopentenylation of adenosine residue of tRNA by the action of several hydrolase and isopentenyl tRNA transferase, which is the product of the *miaA* gene. CBMB20 has two *miaA* genes (MOC_5190 and MOC_5911) and there is a report that *miaA* is critical for the production of zeatin from *Methylobacterium* [96], [97]. Auxin and cytokinin may evoke the plant cell-wall loosening and can cause the nutrient leakages from the host plants as well as activation of plant growth [13], [98].

The presence of ACC deaminase has been studied in various plant growth-promoting bacteria [99]–[101], which may be involved in modulating the associated ethylene signalling pathway. Our previous study demonstrated that *Methylobacterium* can utilize ACC, a precursor of ethylene, as a nitrogen source by the action of ACC deaminase, which implies that *Methylobacterium* can reduce the ethylene levels [22], [28]. ACC deaminase-producing microorganisms are known to be beneficial to the plants because the high levels of ethylene inhibit the growth of the plants [93]. The gene *accD* (MOC_1898) is present in the CBMB20 genome, which confirms previous studies that the strain is able to metabolize ACC [22] as a way to moderate the stress ethylene response by it is canola seedlings.

Formation of vitamin B_12_ by methylotrophs and its growth-promoting effect on potato, tobacco plants and bryophytes have already been described [102]. In addition, CBMB20 produces vitamin B_12_ [20], a cobalt-containing cofactor that has been suggested to stimulate plant development. The genome has clusters of genes *cobPOQD* (MOC_1844 to MOC_1846, MOC_1850), *cobF* (MOC_1916), *cobTS* (MOC_2383 and MOC_2384), *cobWNGHIJKLEMB* (MOC_3494 to MOC_3504) (Figure S3), which should be involved in vitamin B_12_ biosynthesis. The *icuA* gene that encodes an outer membrane cobalt uptake protein [103] was also present in the chromosome (MOC_4703), whereas *icuB* encoding a periplasmic cobalt binding protein was not found.

Pyrroloquinoline quinone (PQQ), a cofactor of several kinds of enzymes, is known to enhance the growth of several organisms including the plants [93]. PQQ is a prosthetic group of methanol dehydrogenases and its biosynthesis is up-regulated in *Methylobacterium* grown in a methanol containing medium [104]. CBMB20 has *pqqA* (MOC_6034), *pqqECB* (MOC_0672 to MOC_0674), and *pqqFG* (MOC_2200 and MOC_2201) encoding PQQ biosynthesis proteins. One interesting feature is that some completely sequenced *Methylobacterium* species have the *pqqABC/DE* genes as an operon, but in other species the *pqqA* gene is present separately. CBMB20 has the *pqqA* gene 930 kb apart from the *pqqBC/DE* operon.

#### Phytofertilization

Low levels of soluble phosphate are known to limit the growth of plants and microorganisms. In soil, most of the phosphate exists in insoluble forms which cannot be easily absorbed by plants. The rhizosphere microorganisms can solubilize these insoluble phosphates to the form that is available for plants to uptake [93]. Therefore, the phosphate-solubilizing ability of rhizobacteria is important for increasing the plant biomass. 20 to 80% of insoluble phosphates in soil are the organic phosphate [105]. There are three kinds of microbial enzymes that can solubilize organic phosphate. The first is non-specific acid phosphatase, which releases phosphate from phosphoric ester or phosphoric anhydride. The second is phytase, which releases phosphate from phytic acid. Phytic acid is also called *myo*-inositol hexakisphosphate and is a major storage form of phosphate in seeds and pollens. Indeed, CBMB20 forms a clear halo in a plate assay with phytic acid as a sole phosphate source (Figure S4). The third is C-P lyase or phosphonatase, which releases phosphate from organophosphonates [105]. *M. oryzae* has a gene encoding an acid phosphatase (MOC_4804), two genes encoding phytase (MOC_1114 and MOC_5789), and the *phn* operon encoding the C-P lyase system (MOC_4396 to MOC_4408).

Sulfur is a major essential nutrient for all organisms and is important for increasing the quality and yield of crops. However, most of the sulfur in soil is in the reduced inorganic form, which cannot be easily absorbed by plants. This reduced inorganic sulfur can be oxidized by several kinds of enzymes produced by rhiozobacteria. The sulfur-oxidizing bacteria can oxidize hydrogen sulfide, sulfur, sulfite, thiosulfate and various polythionates to sulphate that is the form available for plants. In addition, this sulfur oxidation is important for improvement of soil fertility as well as sulfur cycle. Moreover, the acidity of soil caused by sulfur oxidation is known to improve the solubility of the micronutrients such as N, P, K, Mg and Zn [106].

*M. oryzae* is known to directly oxidize thiosulfate to sulphate without production of S_4_ intermediates such as sulfur, sulfite, tetrathionate, trithionate and polythionate, and the enzyme activities of rhodanese and sulfite oxidase were confirmed that are participated in the S_4_I pathway [107], [108]. CBMB20 has three genes encoding rhodanese (MOC_2374, MOC_2875 and MOC_4345) and two genes encoding sulfite oxidase (MOC_0378 and MOC_1641). On the downstream of a rhodanese gene, the *soxZ* gene (MOC_2375) was detected, but not the other *sox* genes. The sulfur oxidation pathway of *Methylobacterium* is species specific [109] and some of *sox* genes were detected in *M. extorquens*, *M. nodulans* and *Methylobacterium* sp. 4–46 among the genome sequencing completed *Methylobacterium* spp.

### Comparison among Completely Sequenced *Methylobacterium* Species

#### Three genomic groups among *Methylobacterium* spp

**Figure 3:**
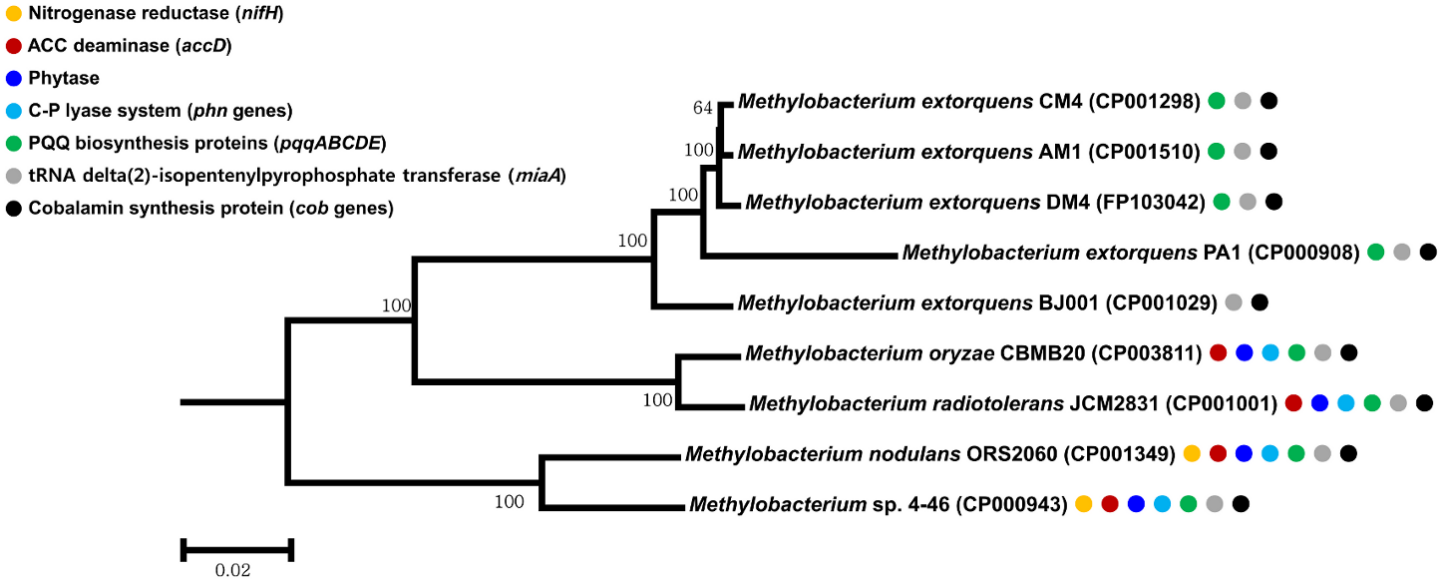
Phylogenetic tree of the members of *Methylobacterium* whose genome sequences have been determined. The amino-acid sequences of 36 broadly conserved proteins were concatenated and aligned with MUSCLE, from which a neighbor-joining tree was constructed using MEGA 5. *Methylocella silvestris* BL2 (CP001280) was used as the out-group. Colored circles indicate the kinds of proteins that are known to promote plant growth.

A phylogenetic tree of all sequenced *Methylobacterium* spp. constructed using broadly conserved 36 proteins [41] revealed that the genus *Methylobacterium* can be divided into three groups (Figure 3). Each of these three groups has distinguishable features. For example, *M. nodulans* and *Methylobacterium* sp. 4–46 mainly exist in the rhizosphere, and are the only species that can fix atmospheric nitrogen with the presence of *nif* genes. *M. extorquens* do not possess the ACC deaminase gene, phytase and the C-P lyase system. Moreover, in the genome of *M. extorquens* BJ006, the *pqqA* gene which encodes a small peptide acting as a precursor [110] was not detected. *M. oryzae* and *M. radiotolerans*, not having the nitrogen-fixing genes, have many plant growth promotion-related genes including the *accD* gene, two genes encoding phytase, PQQ biosynthesis genes, the *miaA* gene, cobalamin-biosynthetic genes, and the C-P lyase system. Average nucleotide identity based on BLAST was higher than 83% between the species within same group, but lower than 78% between the species in the other groups (Table S3).

#### COG and subsystem comparison

**Figure 4:**
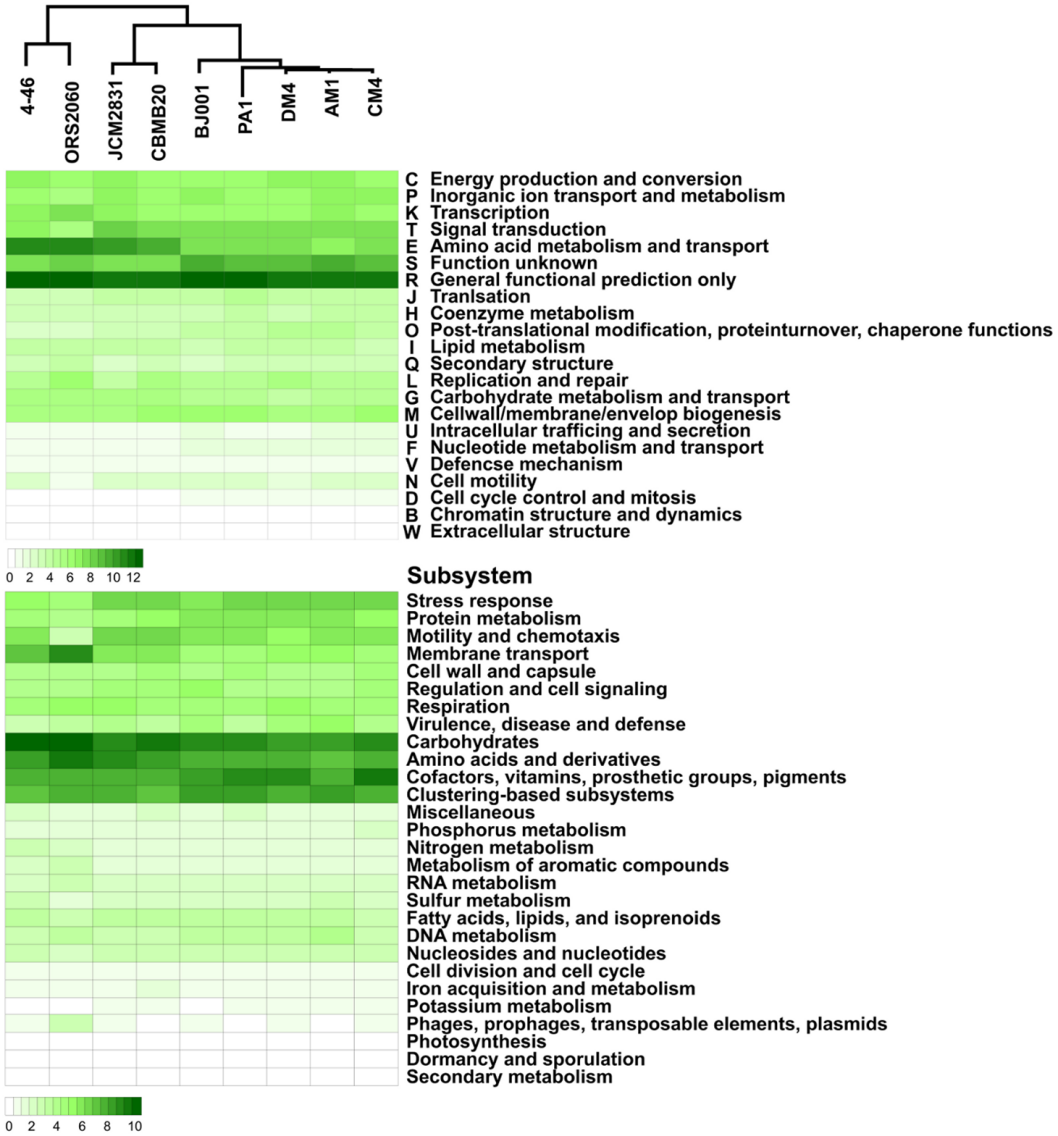
Comparison of the COG and subsystem assignments among completely sequenced *Methylobacterium* species. Color key indicates the relative abundance (Table S4). Heat maps were constructed using R script.

The comparison results from the Clusters of Orthologous Groups (COG) classification [111] demonstrate the striking differences among the groups (Figure 4 and Table S4). Two phyllosphere groups that contain *M. oryzae, M. radiotolerans* and *M. extorquens* have more genes for signal transduction mechanisms (T) and cell motility (N) than the rhizosphere group that contain *M. nodulans* and *Methylobacterium* sp. 4–46. On the other hand, the rhizosphere group has more genes for secondary metabolites biosynthesis, transport and catabolism (Q). One interesting feature is that *M. extorquens* DM4, PA1, and BJ001 have the genes for amino-acid transport and metabolism (E) and carbohydrate metabolism and transport (G) with lower proportions than the other strains of completely sequenced *Methylobacterium*. In fact, *M. extorquens* is known to have the narrowest multi-carbon substrate breath [112]. The subsystem comparison results indicate that *M. nodulans* and *Methylobacterium* sp. 4–46 have fewer genes for ‘motility and chemotaxis’ and ‘stress response’. Likewise with COG comparison, *M. oryzae* and *M. radiotolerans* have more genes for ‘carbohydrates’ and ‘amino acids and derivatives’ than *M. extorquens*. In particular, *M. oryzae* has more genes for ‘iron acquisition and metabolism’ than the others. On the other hand, *M. nodulans* and *Methylobacterium* sp. 4–46 have more genes for ‘membrane transport’, ‘metabolism of aromatic compounds’ and ‘phages, prophages, transposable elements, plasmids’.

#### Core genome and pan-genome

**Figure 5:**
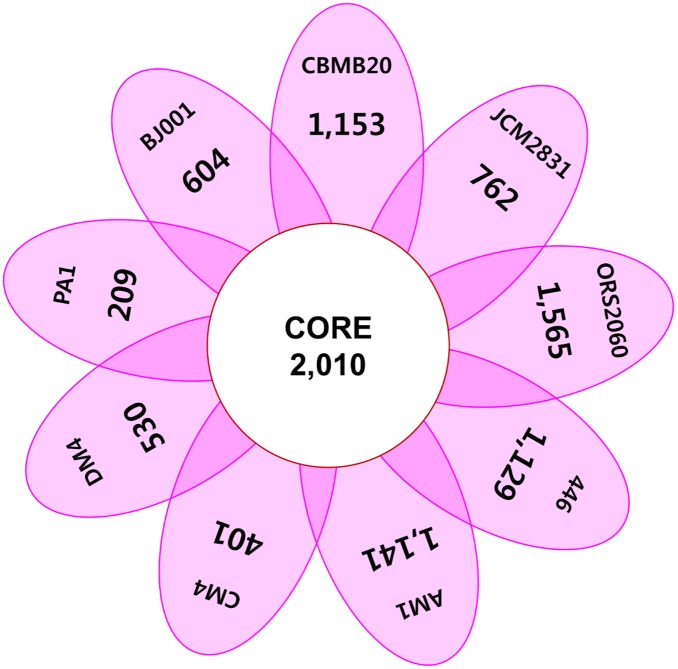
Flower plot of the core genome and strain-specific genes of genome-sequenced *Methylobacterium* strains. The number in the center circle indicates the core genes of completely sequenced *Methylobacterium* strains and the numbers in the floral leafs indicate the number of strain specific CDSs, respectively.

The core genome and the pan-genome of *Methylobacterium* species respectively consist of 2,010 genes and 14,674 genes (Figure 5 and Table 2). The number of *M. oryzae*-specific CDSs is 1,153 that are 17.73% of the total CDSs. The result of the COG and subsystem comparison for strains specific CDSs showed that *M. oryzae* has higher proportion of genes for COG category ‘amino acid metabolism and transport (E)’, ‘lipid metabolism (I)’ and ‘post-translational modification, protein turnover, chaperone function (O)’ and for subsystem category ‘membrane transport’, ‘stress response’ and ‘iron acquisition and metabolism’ than the other strains (Table 3).

**Table:** **Table 2.** Number of shared genes and the pan-genome of *Methylobacterium* species.

Strain	Number of total CDSs	Number of shared CDSs	Number of shared groups*	Pan-genome†
**CBMB20**	6,501	5,348		
**JCM2831**	6,431	5,669		
**ORS2060**	8,308	6,743		
**4–46**	6,692	5,563		
**AM1**	6,205	5,064	7,180	14,674
**CM4**	5,516	5,115		
**DM4**	5,735	5,205		
**PA1**	4,829	4,620		
**BJ001**	5,365	4,761		

*, Shared groups are the CDS groups that encoded by at least two strains.

† , Pan-genome is the sum of the number of shared groups and all strain specific CDSs in Figure 5.

**Table 3:** COG and subsystem comparison of strain-specific CDSs.

(A) COG									
	CBMB20	JCM2831	ORS2060	4–46	AM1	CM4	DM4	PA1	BJ001
**Coverage** *	8.24	16.27	20.38	22.67	10.25	12.22	9.06	14.35	17.72
**S**	17.89	13.71	17.55	12.89	11.97	18.37	12.50	30.00	21.50
**R**	16.84	23.39	19.75	19.14	12.82	14.29	6.25	13.33	8.41
**L**	14.74	3.23	5.02	6.25	12.82	14.29	22.92	13.33	10.28
**M**	10.53	8.06	5.02	10.16	2.56	12.24	2.08	20.00	6.54
**E**	8.42	5.65	6.90	8.20	3.42	4.08	0.00	3.33	4.67
**K**	6.32	6.45	4.70	4.30	12.82	4.08	12.50	3.33	6.54
**C**	4.21	2.42	2.82	6.64	5.13	4.08	4.17	6.67	9.35
**I**	4.21	0.81	3.13	1.56	1.71	0.00	0.00	3.33	1.87
**O**	4.21	3.23	3.45	1.56	4.27	4.08	4.17	0.00	3.74
**G**	2.11	4.03	7.84	8.20	0.85	6.12	6.25	0.00	0.93
**P**	2.11	4.03	5.64	6.25	7.69	2.04	6.25	0.00	4.67
**Q**	2.11	4.03	6.90	4.30	4.27	6.12	2.08	0.00	0.93
**H**	2.11	2.42	0.94	1.95	2.56	6.12	0.00	3.33	1.87
**V**	2.11	1.61	0.94	1.17	1.71	4.08	8.33	0.00	1.87
**T**	1.05	4.84	2.19	2.34	5.13	0.00	6.25	0.00	2.80
**J**	1.05	0.81	0.94	0.78	0.00	0.00	0.00	0.00	0.00
**U**	0.00	6.45	2.82	1.95	4.27	0.00	2.08	0.00	9.35
**D**	0.00	1.61	1.25	1.17	0.85	0.00	0.00	3.33	0.93
**F**	0.00	0.81	0.63	0.78	0.85	0.00	4.17	0.00	2.80
**N**	0.00	1.61	1.25	0.39	4.27	0.00	0.00	0.00	0.93
**W**	0.00	0.81	0.31	0.00	0.00	0.00	0.00	0.00	0.00

(B) Subsystem									
	CBMB20	JCM2831	ORS2060	4–46	AM1	CM4	DM4	PA1	BJ001
**Coverage** *	5.20	15.88	15.08	21.08	9.03	9.23	7.74	7.18	12.09
**Membrane transport**	18.33	6.61	12.71	7.56	16.50	5.41	7.32	13.33	9.59
**DNA metabolism**	16.67	4.96	5.93	5.46	11.65	5.41	24.39	13.33	5.48
**Motility and chemotaxis**	6.67	7.44	3.81	17.65	2.91	2.70	2.44	0.00	1.37
**Carbohydrates**	6.67	2.48	9.32	10.92	0.97	24.32	7.32	0.00	5.48
**Stress response**	6.67	4.13	2.12	2.10	1.94	5.41	2.44	0.00	2.74
**Regulation and cell signaling**	5.00	4.13	2.97	2.94	4.85	8.11	4.88	6.67	9.59
**RNA metabolism**	5.00	3.31	5.51	2.94	2.91	0.00	0.00	6.67	0.00
**Metabolism of aromatic compounds**	5.00	0.83	4.24	1.26	0.97	2.70	2.44	0.00	1.37
**Cell wall and capsule**	3.33	3.31	2.54	5.04	0.97	5.41	2.44	6.67	2.74
**Cofactors, vitamins, prosthetic groups, pigments**	3.33	4.96	5.93	4.62	5.83	5.41	0.00	6.67	5.48
**Protein metabolism**	3.33	3.31	2.12	1.68	5.83	2.70	2.44	13.33	4.11
**Virulence, disease and defense**	3.33	12.40	2.54	1.26	9.71	0.00	9.76	0.00	10.96
**Iron acquisition and metabolism**	3.33	0.00	0.85	0.42	0.00	0.00	0.00	0.00	0.00
**Clustering-based subsystems**	1.67	8.26	14.41	7.98	8.74	10.81	4.88	6.67	2.74
**Amino acids and derivatives**	1.67	13.22	4.24	4.20	1.94	5.41	2.44	13.33	1.37
**Respiration**	1.67	4.13	3.39	4.20	1.94	8.11	7.32	0.00	12.33
**Miscellaneous**	1.67	2.48	1.27	2.52	0.97	0.00	0.00	0.00	0.00
**Sulfur metabolism**	1.67	3.31	1.27	2.52	2.91	0.00	2.44	6.67	2.74
**Phages, prophages, transposable elements, plasmids**	1.67	4.96	4.24	0.42	4.85	5.41	12.20	0.00	17.81
**Potassium metabolism**	1.67	0.00	1.27	0.42	1.94	0.00	0.00	6.67	0.00
**Dormancy and sporulation**	1.67	0.83	0.42	0.00	0.97	0.00	0.00	0.00	0.00
**Nitrogen metabolism**	0.00	0.00	0.85	4.20	0.00	2.70	0.00	0.00	0.00
**Cell division and cell cycle**	0.00	2.48	2.54	2.52	5.83	0.00	2.44	0.00	1.37
**Nucleosides and nucleotides**	0.00	1.65	0.85	2.10	0.97	0.00	2.44	0.00	2.74
**Fatty acids, lipids, and isoprenoids**	0.00	0.83	2.54	2.10	3.88	0.00	0.00	0.00	0.00
**Phosphorus metabolism**	0.00	0.00	1.69	1.68	0.00	0.00	0.00	0.00	0.00
**Photosynthesis**	0.00	0.00	0.00	1.26	0.00	0.00	0.00	0.00	0.00

The numbers indicate the relative abundance of COG and subsystem assigned genes.

*, Coverage means the proportion of COG and subsystem assigned CDSs in strain specific CDSs.

#### The *mxa* gene cluster

Methylotrophs are of polyphyletic origin [113], and the *mxa* gene cluster which is one of the major methylotrophy gene clusters is highly conserved among methylotrophs, including *M. oryzae*, that even belong to the *Gamma-* or *Betaproteobacteria*. This gene cluster is located on a plasmid in *M. nodulans*, whereas it is absent in *Methylobacterium* sp. 4–46. In many species, though not in *M. oryzae*, transposase genes exist in the neighborhood of the gene cluster. Therefore, this methylotrophy island could be considered as a transposable metabolic island, a fitness island, or a utility island.

## Conclusions

**Figure 6:**
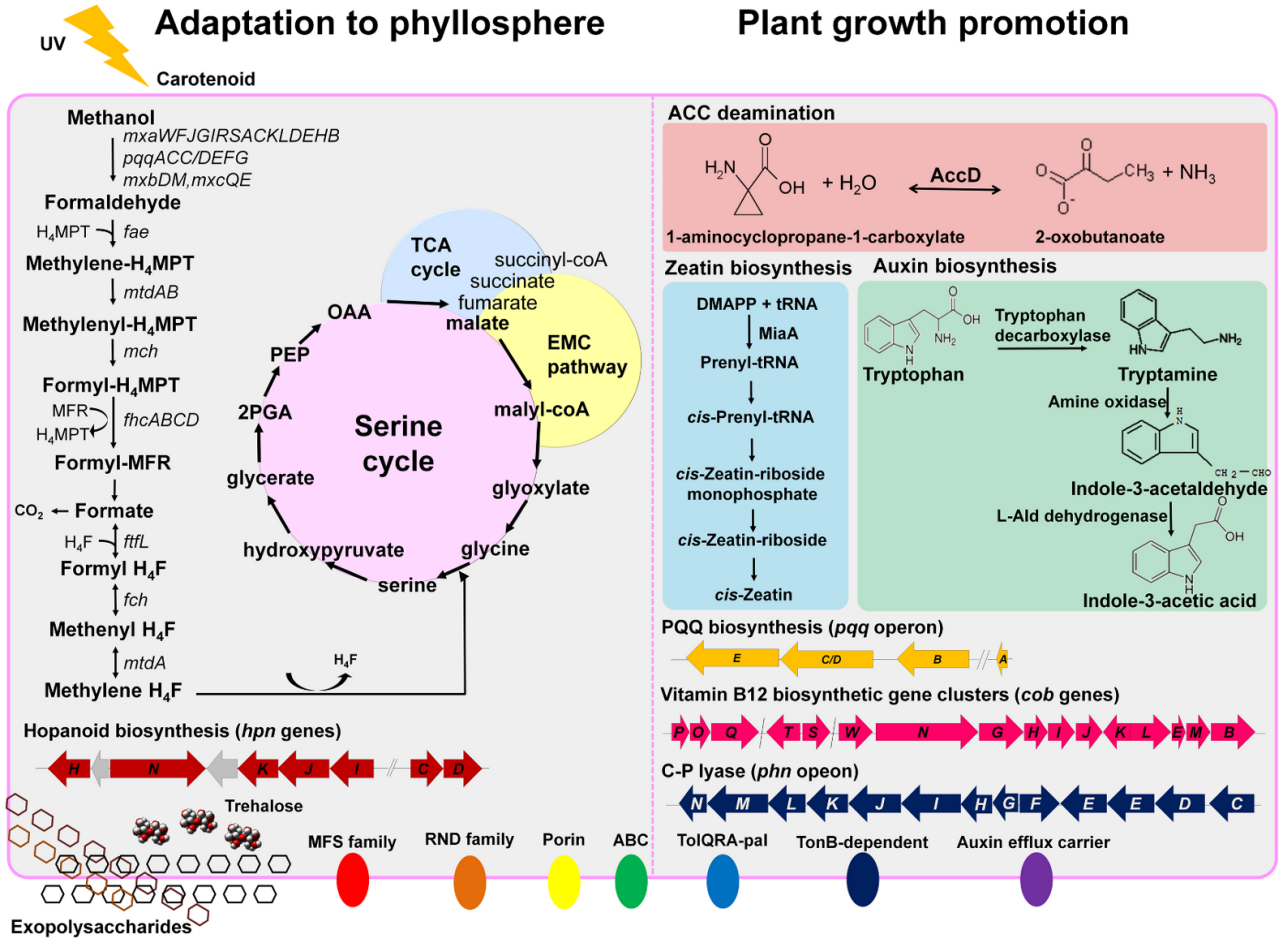
Methylotrophy and adaptation to the phyllosphere. Its genome contains an assortment of genes that may contribute to adaption to the phyllosphere, interaction with plants, and promotion of plant growth. H_4_F, tetrahydrofolate; H_4_MPT, tetrahydromethanopterin; MFR, methanofuran; PGA, phosphoglycerate; PEP, phosphoenolpyruvate; OAA, oxaloacetate; EMC, ethylmalonyl-CoA pathway; TCA, tricarboxylic acid cycle; ACC, 1-aminocyclopropane-1-carboxylate; AccD, 1-aminocyclopropane-1-carboxylate deaminase; DMAPP, dimethylallyl diphosphate; MiaA, tRNA isopentenylpyrophosphate transferase; PQQ, pyrroloquinoline quinone.

In the phyllosphere, there are diverse microorganisms including bacteria, fungi, yeast, and even protozoa and nematodes [13], [114]. C_1_ assimilation, a major characteristic of *Methylobacterium*, may provide an advantage in this highly competitive environment [8]. One of the methylotrophy islands, the *mxa* gene cluster is highly conserved and may be part of a mobile metabolic island. We detected many genes that enable *M. oryzae* to thrive at the surface of plants. *M. oryzae* has more genes encoding motility- or signaling-related proteins and the ECF family sigma factors than other genera in the *Rhizobiales*. This implies that *M. oryzae* can more efficiently respond to cues from extracellular environments, and colonize the phyllosphere. We also identified several kinds of genes related to plant growth promotion in the *M. oryzae* genome that are responsible for producing auxin, cytokinin, ACC deaminase, and the C-P lyase system. While many microbial bioinoculants are currently targeted to the rhizosphere and focused on biofertilization, top-dressing on the surface of the plant leaves may open another possibility for application of bioinoculants. Based on the genome information and experimental evidence, *M. oryzae* can be a good candidate as a bioinoculant targeted to the phyllosphere and focused on phytostimulation. In conclusion, through the genome project of *M. oryzae* CBMB20, we provide the genomic background for adaptation of *Methylobacterium* in the aerial parts of plants and confirm the existence of a number of plant growth promotion-related genes (Figure 6).

## Supporting Information

Figure S1 **Photosynthesis gene clusters in** ***Methylobacterium*** **and other genera in** ***Rhizobiales.***
(DOCX)

Figure S2 **Copper resistance of CBMB20.**
(DOCX)

Figure S3 **Vitamin B_12_ biosynthetic gene clusters in** ***Methylobacterium*** **species.**
(DOCX)

Figure S4 **Phosphate solubilization of CBMB20.**
(DOCX)

Table S1 **Genomic islands of** ***Methylobacterium oryzae*** **CBMB20.**
(XLSX)

Table S2 **Methylotrophy islands of** ***Methylobacterium oryzae*** **CBMB20.**
(XLSX)

Table S3 **Average nucleotide identity based on BLAST between the strains within the genome-sequenced** ***Methylobacterium.***
(XLSX)

Table S4 **Comparison of COG and subsystem assignments in** ***Methylobacterium.***
(XLSX)
