# Supplementary figures and images for: Genome Information of Methylobacterium oryzae, a Plant-Probiotic Methylotroph in the Phyllosphere
Source: PLoS One. 2014 Sep 11;9(9):e106704. doi: 10.1371/journal.pone.0106704 (PMC4161386; doi:10.1371/journal.pone.0106704)

**Figure S1.** Photosynthesis gene clusters in *Methylobacterium* and other genera in *Rhizobiales*.


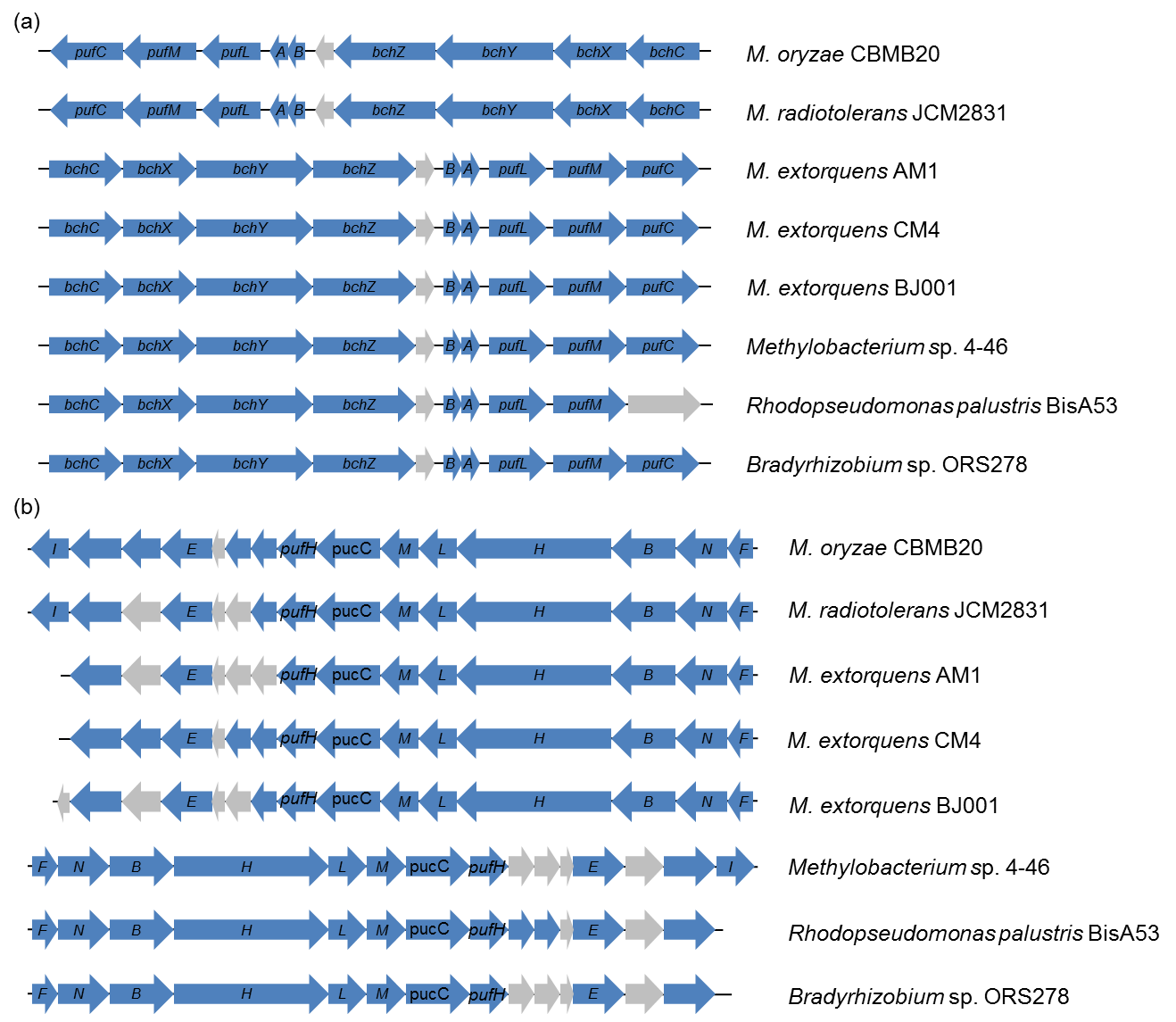

Supplement: Figure S1 — Photosynthesis gene clusters in Methylobacterium and other genera in Rhizobiales. (DOCX) [file pone.0106704.s001.docx]

**Figure S3.** Vitamin B12 biosynthetic gene clusters in *Methylobacterium* species.


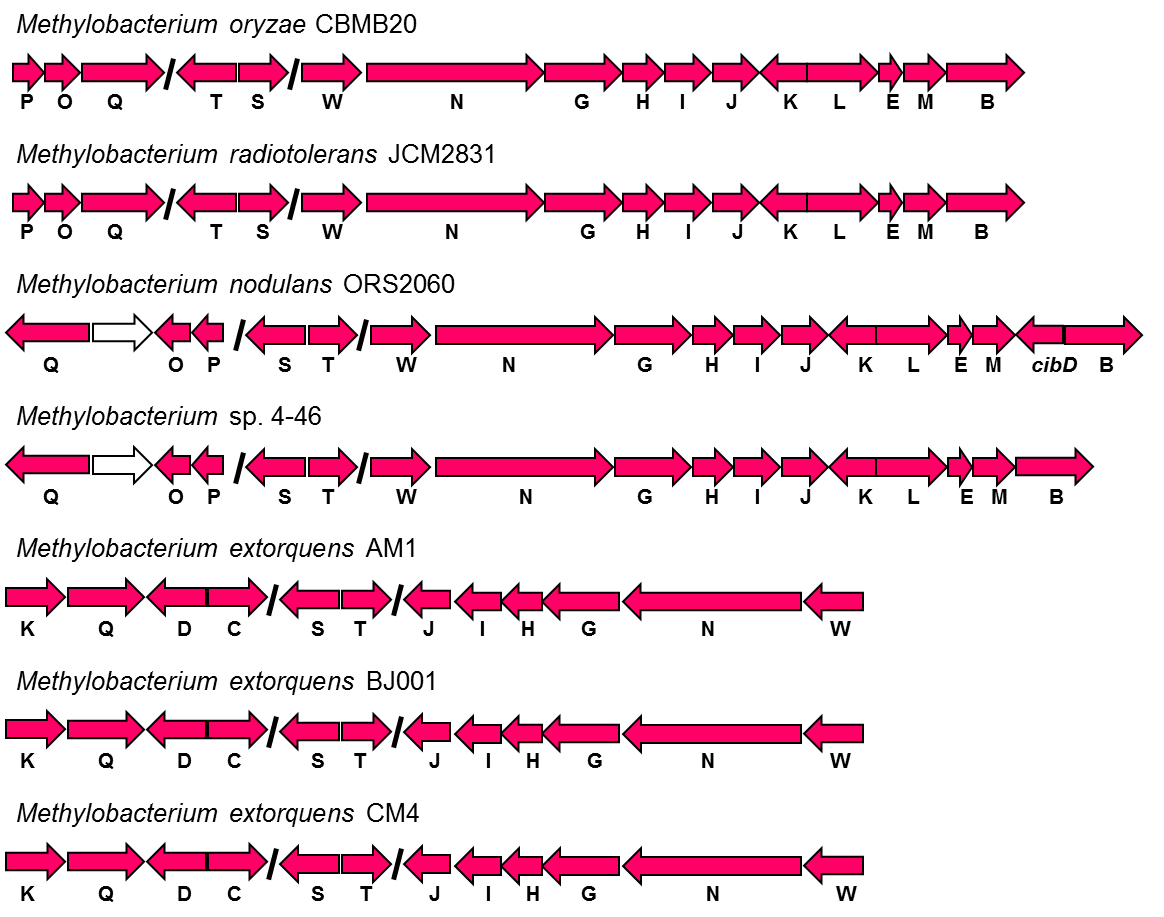

Supplement: Figure S3 — Vitamin B12 biosynthetic gene clusters in Methylobacterium species. (DOCX) [file pone.0106704.s003.docx]
