# Supplementary material for: Genome Information of Methylobacterium oryzae, a Plant-Probiotic Methylotroph in the Phyllosphere
Source: PLoS One. 2014 Sep 11;9(9):e106704. doi: 10.1371/journal.pone.0106704 (PMC4161386; doi:10.1371/journal.pone.0106704)

**Figure S2. Copper resistance of CBMB20**. Copper resistance was tested with L-broth containing various concentrations of copper (CuSO_4_∙5H_2_O; 0.1~4 mM). -; negative control, +; positive control


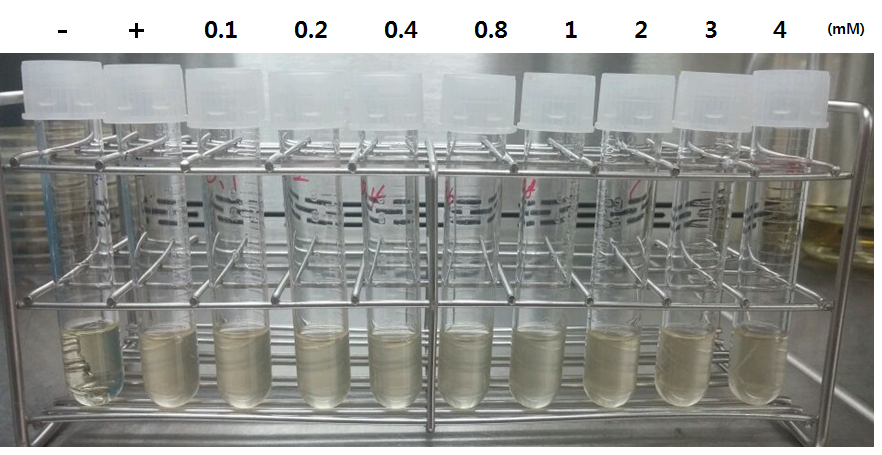

Supplement: Figure S2 — Copper resistance of CBMB20. (DOCX) [file pone.0106704.s002.docx]
