# Supplementary material for: Genome Information of Methylobacterium oryzae, a Plant-Probiotic Methylotroph in the Phyllosphere
Source: PLoS One. 2014 Sep 11;9(9):e106704. doi: 10.1371/journal.pone.0106704 (PMC4161386; doi:10.1371/journal.pone.0106704)

**Figure S4.** **Phosphate solubilization of CBMB20.** (A) *M. oryzae* CBMB20 shows the phytase activity on an ammonium mineral salt agar plate containing phytic acid. (B) CBMB20 cannot solubilize the mineral phosphate on an ammonium mineral salt agar plate containing calcium phosphate.

(A) Organic phosphate solubilization


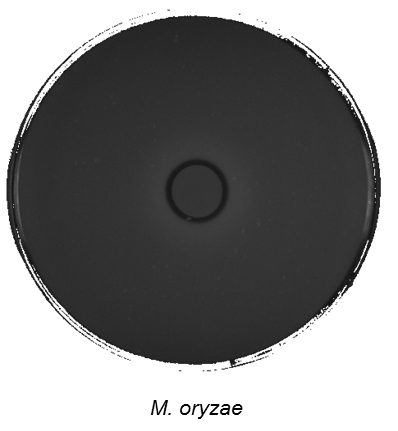


(B) Inorganic phosphate solubilization


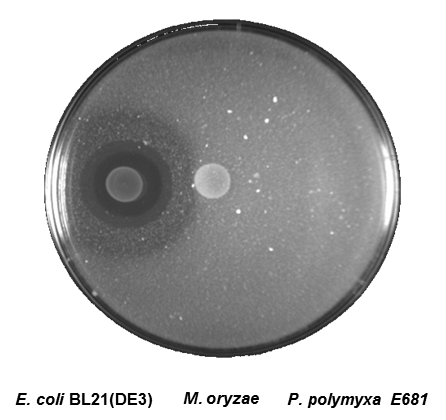

Supplement: Figure S4 — Phosphate solubilization of CBMB20. (DOCX) [file pone.0106704.s004.docx]
